# Major copy proportion analysis of tumor samples using SNP arrays

**DOI:** 10.1186/1471-2105-9-204

**Published:** 2008-04-21

**Authors:** Cheng Li, Rameen Beroukhim, Barbara A Weir, Wendy Winckler, Levi A Garraway, William R Sellers, Matthew Meyerson

**Affiliations:** 1 Departments of Biostatistics and Computational Biology, Dana-Farber Cancer Institute and Harvard School of Public Health, 3 Blackfan Circle, Boston, MA 02115, USA; 2 Deparment of Medical Oncology, Dana-Farber Cancer Institute and Harvard Medical School, 44 Binney St., Boston, MA 02115, USA; 3 Broad Institute of Harvard and MIT, 320 Charles Street, Cambridge, MA 02141, USA; 4 Novartis Institutes of BioMedical Research, 250 Massachusetts Avenue, Cambridge, MA 02139, USA

## Abstract

**Background:**

Single nucleotide polymorphisms (SNPs) are the most common genetic variations in the human genome and are useful as genomic markers. Oligonucleotide SNP microarrays have been developed for high-throughput genotyping of up to 900,000 human SNPs and have been used widely in linkage and cancer genomics studies. We have previously used Hidden Markov Models (HMM) to analyze SNP array data for inferring copy numbers and loss-of-heterozygosity (LOH) from paired normal and tumor samples and unpaired tumor samples.

**Results:**

We proposed and implemented major copy proportion (MCP) analysis of oligonucleotide SNP array data. A HMM was constructed to infer unobserved MCP states from observed allele-specific signals through emission and transition distributions. We used 10 K, 100 K and 250 K SNP array datasets to compare MCP analysis with LOH and copy number analysis, and showed that MCP performs better than LOH analysis for allelic-imbalanced chromosome regions and normal contaminated samples. The major and minor copy alleles can also be inferred from allelic-imbalanced regions by MCP analysis.

**Conclusion:**

MCP extends tumor LOH analysis to allelic imbalance analysis and supplies complementary information to total copy numbers. MCP analysis of mixing normal and tumor samples suggests the utility of MCP analysis of normal-contaminated tumor samples. The described analysis and visualization methods are readily available in the user-friendly dChip software.

## Background

A normal human cell has 23 pairs of chromosomes. For each of the autosomal chromosomes (1 to 22), there are two copies of homologous chromosomes inherited respectively from the father and mother of an individual. However, in a tumor cell, the copy number may be different from two at certain chromosomal regions due to deletion and amplification events. Loss of the contribution of one parent in selected chromosome regions can also happen due to hemizygous deletion or mitotic gene conversion (termed loss of heterozygosity or LOH). When such alterations affect tumor-suppressor genes (TSG) or oncogenes and confer growth advantage to cells, they will be selected in descendant cells and contribute to cancer formation [1]. Identifying such abnormal copy number and LOH regions in tumor samples may thus help to identify cancer-related genes and provide clues about cancer initiation or growth [2,3].

Single nucleotide polymorphisms (SNP) are the most common genetic variations in the human genome. Oligonucleotide SNP microarrays have been developed for high-throughput genotyping of up to 900,000 human SNPs [4,5]. They contain probe sequences complementary to the DNA sequences surrounding the interrogated SNPs. The genomic DNA is amplified, fragmented and labeled, and then hybridized to a SNP array. The scanned array images are analyzed to obtain the genotype calls of all the interrogated SNPs at a high accuracy (>99.3%) [6]. Compared with other experimental techniques, SNP arrays have high throughput and high marker resolution, and they require small amount of DNA per sample (250 ng). They have been utilized in linkage and association studies to identify disease genes [7,8] and in genomics studies to identify LOH and copy number alterations in cancer samples and copy number variation in normal samples [9-11].

The initial methods for analyzing cancer samples using SNP arrays perform copy number and LOH analysis separately [10,12], so each analysis yields genomic alteration information that another analysis may not provide. Recently, allele-specific and parent-specific copy numbers have been developed to utilize allele-specific signals obtained from SNP arrays [13-15]. Allele-specific copy numbers (ASCN) can be estimated from allelic signals at heterozygous SNPs. However, a pair of copy numbers is not straightforward to summarize across multiple samples into a single statistic indicating the excess of genomic alterations. In addition, Hidden Markov Models (HMM) that can successfully smooth total copy number or LOH do not work as efficiently on allele-specific copy numbers due to large number of ASCN states for a pair of copy numbers. In this paper, we defined major copy proportion as a quantity that contains the information difference between the total copy number and ASCN at a locus, and used a HMM algorithm to estimate it from allelic signals. We used 10 K, 100 K and 250 K SNP datasets to demonstrate the method performance and compare it to LOH and copy number analysis.

## Results and Discussion

### Definition of major copy proportion

The major copy proportion (MCP) of a SNP is defined as C_2_/(C_1 _+ C_2_), where C_1 _and C_2 _are the parental copy numbers at this SNP in a sample and C_1 _≤ C_2_. The value of MCP is between 0.5 and 1 by definition, with various values corresponding to different relative proportions of parental copy numbers. The MCP is 0.5 for normal loci or balanced copy alterations, 1 for LOH, and a value between 0.5 and 1 for allelic imbalanced copy number alterations. MCP therefore quantifies allelic imbalance and is a natural extension of LOH analysis. MCP and total copy number (C_1 _+ C_2_) together provide the same amount of information as allele-specific copy numbers, while each of them is a scalar quantity that can be more efficiently estimated and conveniently used in downstream analysis.

We describe here two examples that can benefit from estimating MCP values. First, normal sample contamination in tumors often leads to conservative "No Call" genotypes and intervening LOH or retention calls (see below for specific examples). MCP can better quantify the proportion of normal sample contamination while still identify allelic-imbalanced regions due to LOH. Second, tumors with hyperploidy often contain allelic-imbalanced regions with both parental alleles kept. If the total copy number in such regions is close to the cell ploidy, copy number analysis will reveal a normal relative copy and LOH analysis will show retention. However ASCN or MCP analysis can discover allelic-imbalance as genomic alteration in such regions.

### Hidden Markov Model for estimating MCP

SNP-based LOH or copy number data along a chromosome are locally correlated and HMM is an effective analytic method for such data structure. HMMs have been utilized to analyze array-based copy number changes [12,16-21] and to infer LOH from unpaired tumor samples [22]. Here we used a similar HMM for the MCP inference.

**Figure 1:**
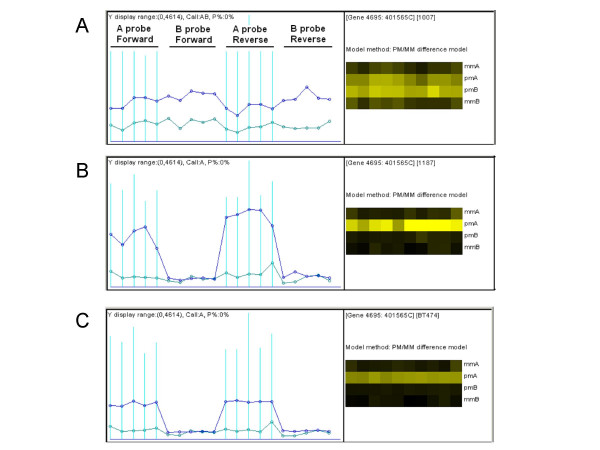
**The probe level data of one SNP**. **(A) **Left: The SNP has 20 probe pairs, whose normalized intensity values in one array are displayed and connected in blue (perfect match or PM) and gray (mismatch or MM) lines. The probe set has probe pairs for both A and B alleles and for the forward and reverse DNA strand. Right: The same probes are displayed in a brightness scale, with the matching A and B probe pairs in the same column. The SNP genotype is called as AB in this sample. **(B) **The probe data of the same SNP in another sample with genotype AA. **(C) **The probe data of the SNP in a third sample with genotype AA. However, the probe signals here are about one half as those in B, presumably corresponding to hemizygous deletion at this SNP position.

The normalized probe intensity values (Figure 1) were used to compute allele-specific SNP signals and raw copy numbers (see "Methods"). We then used HMM to model the MCP correlation of neighboring SNPs on a chromosome. The MCP values to be inferred are between the range of 0.5 to 1 and have 11 states under the default increasing step of 0.05 (comparable to the noise level in our data). The observed data is the raw allele A proportion (RAP), defined as R_A_/(R_A _+ R_B_), where R_A _and R_B _are the raw allelic copy numbers of the two genotype alleles (A and B) at a SNP. For a heterozygous SNP in a sample, RAP should vary by a certain noise level around the unobserved MCP (when A is the major copy allele) or 1 – MCP (when A is the minor copy allele); for a homozygous SNP, RAP is close to 1 for genotype AA and close to 0 for genotype BB, since one of R_A _and R_B _is close to 0. These considerations motivate the function form of the HMM emission distribution (see "Methods").

The HMM with emission and transition distributions specify the joint probability of the unobserved MCP and the observed RAP of all SNPs in a chromosome of a sample. The Viterbi algorithm [23] was then used to obtain the most probable MCP state path as the inferred MCP values. The procedure was run separately for all chromosomes and all samples in a dataset.

### Datasets used

We used several SNP array datasets to illustrate the analysis and visualization methods and to compare the results. (1) 10 K SNP dataset. Zhao et al. [12,24] generated Early Access 10 K SNP array data for 14 breast and lung carcinoma cell lines and their paired normal cell lines, as well as 4 primary lung carcinomas and their paired normals. The array contains 10,043 SNPs with an average resolution of 300 kb. This work is one of the first demonstrations of combined copy number and LOH analysis using SNP arrays. (2) 100 K SNP dataset. Zhao et al. [25,26] generated 100 K SNP array data for 70 primary human lung carcinoma specimens and 31 cell lines derived from human lung carcinomas. 12 unpaired normal samples were used as reference in copy number analysis. The array contains 115,593 SNP with an average resolution of 24 kb. LaFramboise et al. [13] further analyzed this dataset to develop allele-specific copy number analysis and generated allele-specific quantitative PCR (Q-PCR) measurements for selected loci to compare allele-specific copy numbers from Q-PCR and SNP arrays. (3) 250 K cell line dataset. Affymetrix has made freely available a 500 K (consisting two 250 K SNP arrays) SNP dataset consisting of 9 tumor/normal pairs derived from breast and lung cancer cell line [27]. The average marker resolution is 5.8 kb and 85% of the human genome is within 10 kb of a SNP. In this work we used the subset of the 250 K STY array data. (4) 250 K lung tumor dataset. Weir et al. [28] generated 250 K STY SNP array data for 371 primary lung adenocarcinomas and 242 matched normal samples. We used a subset of 45 pairs of normal and tumor samples that are publicly available [29].

### Visualizing MCP

**Figure 2:**
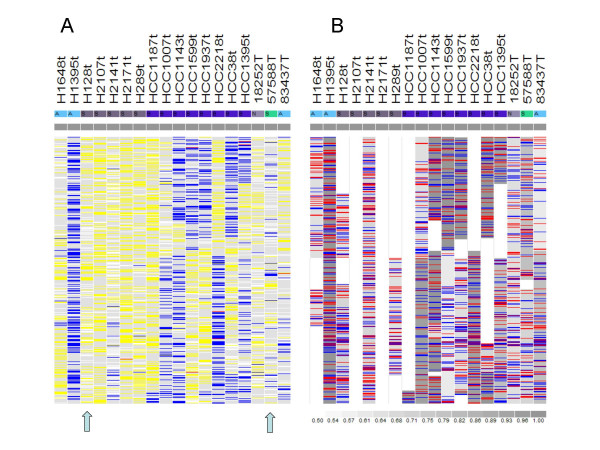
**The LOH and MCP data views of a chromosome**. The tumor samples are displayed on columns and the SNPs are ordered on rows by their chromosome positions. **(A) **Observed LOH calls by comparing normal (N) and tumor (T) genotypes at the same SNP. Yellow: retention (AB in both N and T), Blue: LOH (AB in N, AA or BB in T), Red: Conflict (AA/BB in N, BB/AA or AB in T), Gray: Non-informative (AA/BB in both N and T), White: No Call (No Call in N or T). **(B) **In the inferred MCP data view, the white and gray colors represent inferred MCP levels from 0.5 to 1 according to the color scale. When the inferred major copy allele is estimated to be different from the minor copy allele at a SNP (see "Methods"), the SNP position is colored in red or blue according to the major copy allele (A or B).

The dChip software [30,31] was used to implement the methods and visualize the analysis results. Figure 2 compares the observed LOH view in dChip with the new MCP view using the chromosome 7 of the 10 K SNP dataset. In the MCP view (Figure 2B), different shades of gray correspond to MCP values greater than 0.5, highlighting allelic imbalanced regions. Comparing to the raw LOH calls on the left, MCP discovers more allelic-imbalanced regions that could cause excessive No Calls (sample H128t) or intervening LOH and retention calls (sample 57588T) in genotype-based LOH analysis.

**Figure 3:**
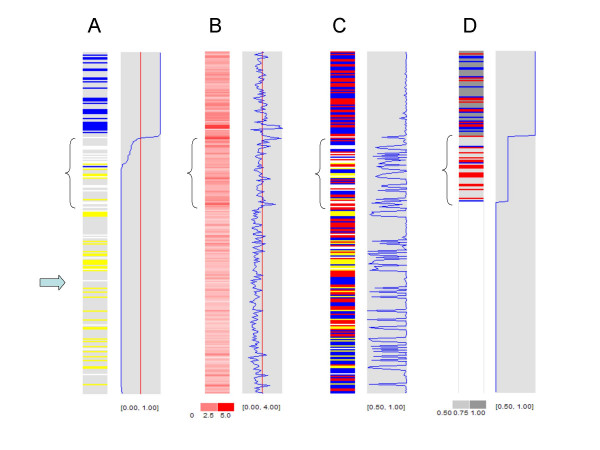
**The LOH, copy number, genotype and MCP data views of chromosome 18 of sample H1395t**. **(A) **LOH from paired analysis, similar to Figure 2A. **(B) **Raw copy numbers. **(C) **Genotype calls. The red, yellow, blue and white colors represent genotype AA, AB, BB and No Call. **(D) **Inferred MCP, similar to Figure 2B. The blue curves displayed on the right in the gray boxes represent: (A) inferred LOH probability, (B) raw copy number, (C) raw major allele proportion (MAP), computed as Max(R_A_, R_B_)/(R_A _+ R_B_), and (D) inferred MCP. Comparing the two curve in C and D, we can visually assess how the HMM infers MCP at regions of mixed homozygous and heterozygous genotypes.

In another example, we compared different SNP data views of a lung cancer cell line with paired normal (sample H1395 from the 10 K dataset). The most interesting region in chromosome 18 is indicated by braces in Figure 3. The tumor sample contains many No Call genotypes in this region (white colors in Figure 3C), and the paired LOH analysis yields intervening retention, LOH and No Calls (Figure 3A). The raw copy numbers of this region center around ploidy or the relative copy number 2 (Figure 3B). The raw major allele proportion curve in Figure 3C reveals that most values are either close to 1 (corresponding to homozygous SNPs) or between 0.6 and 0.7. A likely explanation for these data is that this chromosome region has three copies and the whole genome is near triploid, which is confirmed by spectral karyotyping data [32]. Two copies of this region are from one parent and one copy is from another, creating underlying MCP of two thirds (0.67), close to the inferred MCP value of 0.65 (blue curve in Figure 3D). Interestingly, the chromosome region below this region has retention of heterozygosity, a MCP of 0.5, but copy numbers below ploidy (indicated by arrow in Figure 3). This region most likely has one copy of each parental chromosome, creating copy number decrease from ploidy while retaining the heterozygosity.

Therefore, genotype-based LOH analysis suggests the middle region in Figure 3 to be unusual, but the MCP analysis helps to pinpoint the underlying cause of the abnormality. The MCP result also reveals that the heterozygous SNPs in this region have real genotype AAB or ABB. The standard genotyping algorithm are trained by normal samples [6,33], thus making conservative No Call or incorrect AB or AA/BB call for these complex genotypes and leading to intervening LOH, retention or No Calls in genotype-based LOH analysis. Combining MCP and total copy number, we can thus obtain a more complete understanding of the genomic structure of tumor samples.

### Comparing MCP and LOH

**Figure 4:**
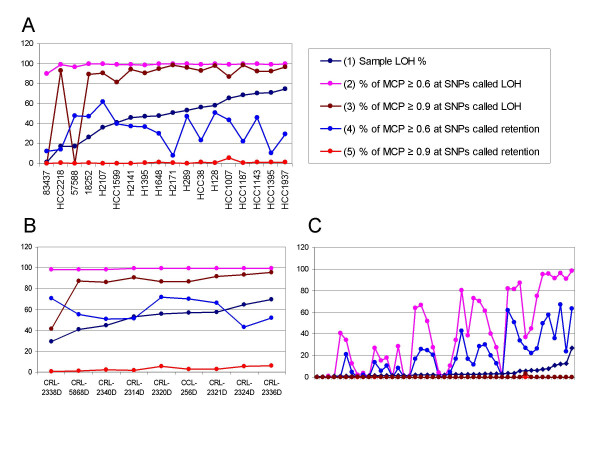
**Comparing LOH and MCP from paired analysis**. The samples are ordered on the X-axis by their sample LOH percentage from paired LOH analysis. (A) 10 K SNP data. (B) 250 K cell line data. (C) 250 K lung tumor data, where the sample names are omitted.

We then used 18 pairs of normal and tumor samples in the 10 K dataset to compare the paired LOH and MCP analysis. 14 of these pairs are normal and tumor cell line samples. SNPs were classified as LOH or retention SNPs based on paired LOH analysis (Figure 2A) and then compared with their MCP values. Figure 4A orders these tumor samples by their increasing sample-wise LOH rates (1.2% to 75%, curve 1). In all the samples, more than 90% LOH SNPs have MCP ≥ 0.6 (curve 2); in all but two samples, more than 80% LOH SNPs have MCP ≥ 0.9 (curve 3). The two samples 83437 and 57588 both have LOH rate below 20% and most of their LOH SNPs are in the intervening LOH and retention regions (the last two columns of Figure 2A). MCP values between 0.6 and 0.9 correctly identified these regions as allelic imbalance rather than LOH. In fact, these two samples are primary tumor samples and could contain normal sample contamination [12], which likely cause most LOH areas in pure tumor cells to become allelic imbalance in the normal-contaminated tumor samples. In contrast to the LOH SNPs, the retention SNPs seldom have MCP value ≥ 0.9 in all the samples (curve 5), although in many samples more than 20% of the retention SNPs have MCP value ≥ 0.6 (curve 4). These regions often have copy number alterations that cause allelic imbalance but not LOH, leading to intervening retention and LOH calls (the last three columns of Figure 2A).

**Figure 5:**
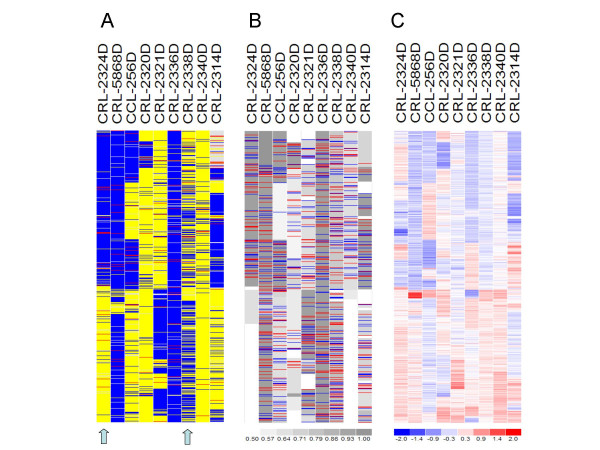
**The LOH, MCP and copy number data of chromosome 1 of the 250 K cell line data**. (A) The LOH calls from paired normal and tumor analysis. (B) The inferred MCP values. (C) The copy number of tumor samples is displayed in log2 ratio scale relative to the normal copy of 2.

We also made similar comparison using the two 250 K datasets. Figure 4B shows the same percentages as Figure 4A for the 250 K cell line dataset of 9 sample pairs. All percentages have similar performance as the 10 K data. The percent of MCP ≥ 0.9 at paired LOH calls (curve 3) is low at the sample CRL-2338D. Inspecting the paired LOH calls in this sample reveals that most LOH in them are intervened with retention calls (Figure 5A), indicating allelic-imbalance rather than LOH. In contrast, the MCP analysis inferred smooth and moderate MCP values in this sample (Figure 5B), better discovering the underlying genomic alterations. Figure 4C shows the same comparison percentages for the 250 K lung primary tumor dataset of 45 sample pairs. All samples except one have paired LOH call percentage below 20% (curve 1). This can be due that the LOH events are at a lower frequency in these primary tumors or that the homozygous genotypes in LOH regions of tumor cells are masked by normal sample contamination up to 30% [28]. The portions of MCP ≥ 0.6 among paired LOH or retention calls both drop as paired LOH percentage drops (curve 2 and 4), while the portions of MCP ≥ 0.9 among paired LOH are near zero for all samples (curve 3, which overlays with curve 5), supporting the existence of normal sample contamination in most samples.

In summary, MCP analysis is able to discover real LOH and retention events. It also discriminates allelic-imbalanced regions from LOH through intermediate MCP values between 0.5 and 1, instead of yielding intervening LOH and retention calls.

### Comparing MCP and copy numbers

It is of interest in cancer research whether copy number gains or amplifications are allelic-balanced or imbalanced events, since the genes in the imbalanced events could have a variant or mutant form preferentially amplified [13]. SNP arrays have the advantage of providing both allelic-imbalance and copy number information. We have previously used the 10 K dataset to show that the LOH events can involve copy number deletion, copy-neutral or amplification events, while retention mostly occur at copy-neutral or amplification events [22]. With the inferred MCP as the extension of LOH calls, we now ask how allelic imbalanced events correlate with copy numbers. A visual comparison of LOH, MCP and copy number using the 250 K cell line dataset is in Figure 5. In the p-arm of sample CRL-2324D, LOH events correspond to both copy number amplifications and deletions.

**Figure 6:**
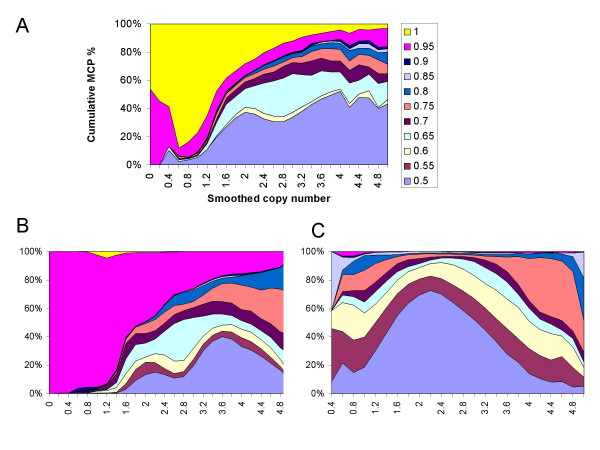
**Stratifying MCP according to copy numbers**. The 5-SNP smoothed copy numbers were scaled to have mode at two copies for each sample, and then were binned into copy number intervals of a width of 0.2. For example, on the X-axis, 0 indicates the copy number bin [0, 0.2] and 2 indicates the copy number bin [2, 2.2]. The cumulative percentages of MCP for the SNPs in a particular copy number bin were displayed on the Y-axis, using all cell line or tumor samples of the 10 K data (A), 250 K cell line data (B), and 250 K lung tumor data (C). In Figure C, the copy interval 0 and 0.2 are not plotted since there are fewer than 20 data points to compute percentages.

We then stratified SNPs by copy number bins and computed the distribution of MCP values at various copy numbers. In the 10 K data (mostly cell lines, Figure 6A) and the 250 K cell line data (Figure 6B), the copy numbers below 1.5 mostly correspond to MCP values ≥ 0.95 (LOH or extreme allelic imbalance). As copy number increases, the percent of "MCP ≥ 0.95" decreases while the percent of "MCP ≤ 0.6" (retention or near allelic balance) increases, indicating that larger copy number gains or amplifications involve less frequently with LOH and more frequently with allelic balanced and imbalanced events. In both 10 K and 250 K cell line data, there is a noticeable drop of allelic-balanced events ("MCP ≤ 0.6") around copy number of 3. The fact that 3 copies can have balanced amplifications is due to that the copy numbers from SNP arrays are not absolute copy numbers but relative to the ploidy of tumor cells (see "Methods"). Similarly, there are SNPs with MCP values close to 0.5 but copy number below 1, since in hyper-diploid samples the real copy number 2 has array-based relative copy number below 2. The peak at copy number 0.4 in Figure 6A is likely due to the small sample variation (4 of 39 data points at the copy bin 0.4 have MCP of 0.5) or inaccurately inferred MCP values.

In contrast, different patterns emerge from the 250 K tumor dataset (Figure 6C). The percent of "MCP ≥ 0.95" is nearly zero at all copy numbers, and the portion of allelic-balanced events has a single peak around copy number of 2 and drops rapidly as copy number goes lower or higher. These could be explained by potentially prevalent normal sample contamination in these primary tumors, which could cause the attenuation of aberrant copy number values toward the normal copy of 2, as well as make the LOH or allelic-imbalanced amplification events in tumor cells to appear less allelic-imbalanced in contaminated tumor, leading to high percent of MCP values between 0.55 and 0.75.

Next we used the 100 K SNP array dataset with allele-specific copy numbers measured by quantitative PCR (Q-PCR) to compare the MCP based on SNP arrays and Q-PCR. We used the same SNPs to design the primers for Q-PCR and to obtain their array-based signals for comparison. Since Q-PCR measures allele-specific copy numbers rather than parent-specific copy numbers, we defined major allele proportion (MAP) as Max(A, B)/(A + B) and used it in the comparison, where A and B are Q-PCR or array-based allelic copy numbers. Table 1 shows that most array-based MCP and the Q-PCR-based MAP values agree within a difference of 0.15 ("PCR MAP" and "Array MCP" columns). The largest difference of 0.44 (bold values in the table) occurs at SNP 589797 in sample S0515T. This SNP has homozygous genotype in the sample (both PCR and array-based MAP values are close to 1), but its multiple neighboring SNPs have heterozygous genotypes and MAP values close to 0.5 (data not shown), which contributes to the final inference of MCP 0.55 at the SNP 589797. Together with an amplified total copy number, we conclude that the DNA at the SNP is about equally amplified for both parental alleles.

**Table 1:** Comparison of MCP based on SNP arrays and major allele proportions (MAP, Max(A, B)./(A+B)) based on allele-specific Q-PCR or array-based allelic signals.

Sample	SNP	Chromo-some	Position(Mb)	PCR allele A Copy	PCR allele B Copy	PCR MAP	Array MAP	Array MCP	Array Copy
S0465T	1894228	3	183.975	25.18	1.68	0.94	0.86	0.8	4.8
S0515T	1894075	3	183.786	2.42	38.37	0.94	0.65	0.8	17.47
HCC827	2568690	7	54.606	135.92	1.97	0.99	0.82	0.8	8.07
H2087	2804962	8	128.906	1.23	6.03	0.83	0.74	0.7	6.05
H2122	2804228	8	128.037	58.46	3.39	0.95	0.98	0.95	6.55
HCC827	2804646	8	128.332	0.06	7.58	0.99	0.9	0.95	9.39
S0515T	589797	12	32.822	0.06	7.12	**0.99**	**0.96**	**0.55**	4.32
H2087	590880	12	33.8	17.32	0.03	1	0.88	0.95	10.42
H2087	611421	12	57.198	4.86	0.17	0.97	0.95	0.8	11.3
HCC1359	1679843	22	19.774	1.03	8.36	0.89	0.82	0.9	8.74

Both SNP arrays and Q-PCR yield allelic-specific copy numbers relative to sample ploidy. "Array Copy" is the array-based total copy number by median smoothing of raw copy numbers with a window size of 5 SNPs. The chromosome positions are based on the UCSC hg16 genome assembly.

### MCP analysis of normal contaminated samples

We next checked how well MCP can address two challenges of applying SNP arrays in cancer genomics: tumor samples frequently lack paired normal samples to perform paired LOH or MCP analysis, and tumor tissue samples often contain normal stromal cell contamination. The HMM emission distributions can flexibly use either paired normal genotypes in paired MCP analysis or population-based normal genotype distribution in tumor-only analysis (see "Methods"). We checked how well the MCP estimated using paired normal and tumor samples agree with the MCP estimated using only tumor samples. In the 10 K dataset, the sample-wise absolute MCP differences between the two methods in the 18 samples range from 0.0006 to 0.025, and the sample-wise standard deviations of the MCP differences range from 0.013 to 0.075. In the 250 K lung tumor dataset, these two differences measures are larger across the 45 tumors, ranging from 0.011 to 0.041 and 0.050 to 0.114 respectively. Visual inspection of the MCP inferred from the 250 K data reveals many small regions that have MCP value ≥ 0.5 in tumor-only analysis but have MCP value of 0.5 in paired analysis. They are caused by stretches of homozygous genotypes that are in linkage disequilibrium, in a similar way as the false positives in tumor-only LOH analysis [22]. By utilizing the genotype dependence of neighboring 5 SNPs in the HMM emission probabilities of tumor-only MCP analysis (see "Methods"), the two differences measures are smaller (ranges are 0.0001 – 0.026 and 0.008 – 0.097). Overall the differences between paired and tumor-only MCP inferences are fairly small compared to the values that MCP can take (0.5 to 1).

**Figure 7:**
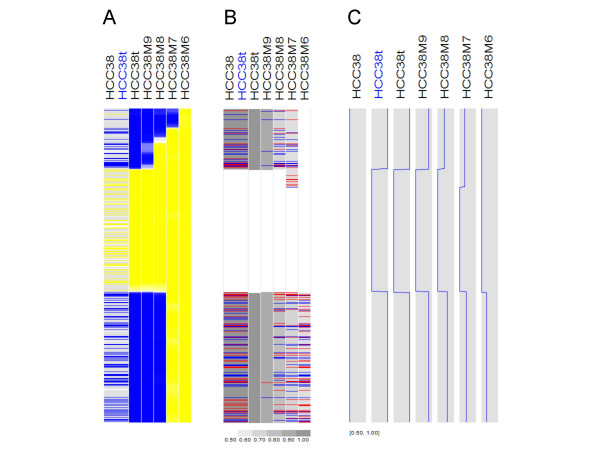
**Comparing LOH and MCP using the 10 K mixing samples**. The inferred LOH (A) and MCP (B, C) are displayed for chromosome 4. In the tumor-only LOH inference (the columns except column 1 in A), the inferred probability of LOH is displayed using a blue (1 – 0.5) to white (0.5) to yellow (0.5 – 0) color scale. See legends of Figure 2 for additional color schemes.

We next asked how much the tumor-only MCP method is tolerant to normal contamination. The 10 K dataset contains a mixing experiment of paired normal and tumor samples [12]. A tumor cell line (HCC38t) was mixed with its paired normal cell line (HCC38) at various proportions and then hybridized to 10 K SNP arrays. In Figure 7, sample HCC38M9 to HCC38M6 are mixture samples with tumor content of 90%, 80%, 70% and 60% respectively. The LOH regions in the pure tumor sample should become allelic-imbalanced regions in the mixture samples. Figure 7 shows a typical example of inferred LOH and MCP data using unpaired analysis (paired analysis for the column labeled with "HCC38t" in blue color). Compared to the LOH data (Figure 7A), MCP analysis better identified the boundaries of the allelic-imbalanced regions in all the mixture samples (Figure 7B). The values of estimated MCP for the LOH regions are less than 1 in the mixture samples (Figure 7C), corresponding to allelic-imbalance created by normal sample contamination. Interestingly, both LOH and MCP analysis performs better for the bottom LOH region than the top LOH region in the mixture samples, due to copy-neutral LOH in the bottom region and hemizygous deletion in the top region (copy number data not shown).

**Figure 8:**
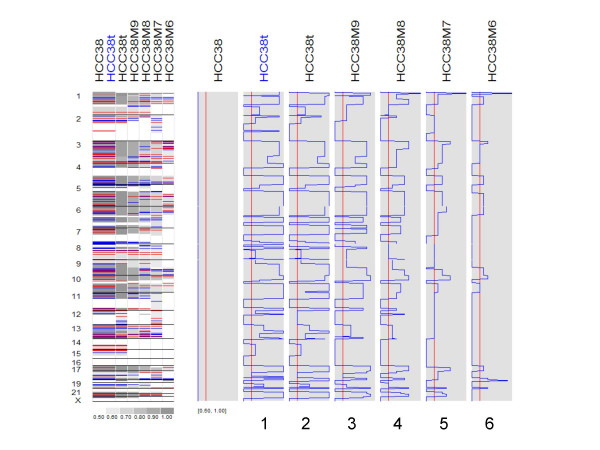
**The genome-wide view of inferred MCP in the mixture samples**. The red vertical lines in the gray boxes represent a MCP threshold of 0.6. See the legend of Figure 2B and 3D for color schemes.

Figure 8 shows the whole-genome MCP values inferred for paired analysis (column 1) and for tumor-only analysis of tumor (column 2) and mixture samples (column 3–6). The MCP patterns largely preserve but MCP values attenuate toward 0.5 as tumor content decreases. If a threshold of "MCP ≥ 0.6" is used to call allele-imbalanced SNPs (red vertical line in the shaded boxes) and we regard paired MCP analysis as the ground truth, at tumor content of 80% (column 4) we could achieve 88.5% for sensitivity and 88.2% for specificity. But at tumor content of 70% (column 5) the sensitivity and specificity dropped to 82.6% and 60.4%. This shows that normal contamination of up to 20% is tolerable when calling allele-imbalanced regions in MCP analysis.

### Major and minor copy alleles

One feature of the MCP algorithm is that it also infers the major and minor copy alleles for SNPs that are heterozygous in normal sample and undergo LOH or allele-imbalance in tumor (see "Methods"). In Figure 7B, a SNP is colored in red or blue for major copy allele A or B if its major copy allele (MCA) can be inferred and is different from the minor copy allele, which is not displayed. In the paired MCP analysis (Figure 7B, column "HCC38t" with blue color), the MCA is inferred to be different from minor copy allele for many SNPs since the normal sample contains information on heterozygous SNPs and LOH in the tumor sample provides information on the kept allele as MCA. In contrast, in the tumor-only MCP analysis (Figure 7B, column "HCC38t" with black color), we can infer two regions of LOH (MCP is 1) as well as MCA, but there is no information on minor copy allele, so no SNP is colored. However, as normal contamination increases from sample HCC38M9 to HCC38M6, the minor copy allele of more and more SNPs can be estimated from the mixing normal sample, so more SNPs are colored to indicate MCA is different from minor copy allele (Figure 7B). These results show that allelic-imbalances (such as those in the mixture samples) can help to distinguish major and minor copy alleles, while LOH or allelic-balanced events can not.

Therefore in tumor-only MCP analysis, normal contamination at low percentage (≤ 20%) can be turned beneficial through MCP analysis. The normal contaminated samples contain the information of both normal sample genotypes and tumor genome alterations (LOH and copy number changes). In the tumor-only MCP analysis, the normal genotype information is utilized in the form of allele-specific raw copy numbers in the HMM emission distributions (see "Methods"). As the result, the allele-imbalanced regions are identified in a similar way to paired LOH analysis via normal-tumor genotype comparison rather than tumor-only LOH inference, which resorts to unrelated reference normal genotypes to distinguish between LOH and homozygous haplotype blocks [22]. The normal contaminated samples also help to provide information on both major and minor copy allele at allelic-imbalanced regions, which can be useful in downstream analysis. If the contamination percentage can be estimated by other experimental measures, the inferred MCP or copy number from normal contaminated samples can be adjusted proportionally to obtain the MCP/LOH and copy number values of the unavailable pure tumor samples. However, efforts should be made to obtain pure tumor samples and their paired normals for separate hybridization whenever possible, as paired MCP analysis can better recover allele-imbalance information than tumor-only MCP analysis of contaminated samples (Figure 8).

### Downstream analysis and related analysis methods

MCP is an extension of LOH and contains complementary information to total copy numbers. Similar to LOH and copy number analysis of a set of tumor samples, a MCP summary score for each SNP may be computed across samples, such as the average MCP value across all samples. Then the chromosome regions can be permuted within samples, and the MCP scores computed from the permuted data can be compared to the original MCP scores to assess the significance of the latter [34]. A composite alteration score using both MCP and total copy number may also be used, such as the proportion of samples with copy > 3 and MCP > 0.65 to capture only allelic imbalanced amplifications.

There are several allele-specific copy number (ASCN) analysis methods for SNP arrays [13,14,16,35]. While total copy number plus MCP provide the same amount of information as allele-specific copy numbers, MCP extends the LOH analysis in a natural way and offers a univariate statistic to capture both LOH and allele-imbalance events. Such univariate quantity is more efficiently estimated and easily used in downstream analyses than a pair of allelic copy numbers. If needed, the total copy number and MCP can be combined to be equal to the analysis using allelic-specific copy numbers. The MCP analysis also reports major and minor copy alleles for allelic comparisons in allele-imbalanced regions. Several of the above ASCN methods also use probe sequence and restriction fragment length to adjust for probe signals to improve signal to noise ratios. Such adjusted raw allelic copy numbers can be conveniently used as the input of the MCP analysis through the dChip software.

Similar to all copy number analysis of SNP arrays, ideally we need paired normal samples for MCP analysis. When such paired samples are not available and an independent set of normal samples are used for reference signals, copy number variations (CNV) in normal samples may confound tumor copy number analysis [36,37]. To address this issue, we have implemented a trimming method. Specifically, we assumed that in reference normal samples, for any SNP at most a certain percent (such as 10%) of the samples have abnormal copy numbers. Then for each SNP, 5% of samples with extreme signals are trimmed from the high and low end of the raw signal distribution and the rest samples are used to compute the signal mean and standard deviations of normal copy numbers at the SNP. This trimming method is designed to accommodate a small amount of CNVs in reference normal samples and has proven useful in copy number analysis with unpaired or limited number of normal samples. The same trimming method can be used to obtain raw allele-specific copy numbers in the MCP analysis.

## Conclusion

In this paper, we have focused on allelic imbalance analysis of tumor samples using SNP arrays. We proposed to estimate major copy proportion, which is an extension of LOH analysis and complements total copy number analysis. HMM is used to bridge unobserved states (MCP) and observed data (allele specific signals) through emission and transition distributions. We compared the inferred MCP with LOH and copy number analysis and demonstrated that MCP performs better than LOH analysis in allelic-imbalanced regions and normal contaminated samples. The major and minor copy alleles can also be inferred at allelic-imbalanced regions by MCP analysis. The described analysis and visualization methods are readily available in the dChip software.

## Methods

### Computing allele-specific raw copy numbers

We use the Invariant Set Normalization method to normalize all the arrays at the probe intensity level to a baseline array with moderate overall intensity [38]. Due to the fact that same amount of DNA sample are hybridized onto arrays and the normalization procedure, the total copy numbers estimated from SNP arrays are relative to sample ploidy. However, the inferred MCP estimates the real MCP in tumor cells, since hybridization and normalization affect the raw signals of both alleles proportionally. We then computed the allele-specific signals for each SNP and sample by applying the PM/MM difference model [39] separately to the probe-level data of A alleles and B alleles of all samples at a SNP probe set (Figure 1).

To obtain allele-specific raw copy numbers for a SNP, the allele-specific signal values of all normal samples (usually ≥ 10, e.g. [16]) in a dataset and their genotypes are first used to estimate allele-specific signal distribution. Specifically, for each SNP, the A allele signal of a genotype AB or half of the A allele signal of a genotype AA are regarded as sample data points from the signal distribution of one copy of A allele and are used to estimate the mean and standard deviation of this distribution. The similar is done for the B allele's signal distribution. When there are fewer than six observed data points to estimate the allele-specific distributions, the total signal (sum of A and B allele signals) of all normal samples will be used to construct allele-independent distribution of one copy [12] and used in place of allele-specific distributions. Finally, the allele-specific signals and standard deviations are divided by the allele-specific means to obtain the allele-specific raw copy numbers (R_A _and R_B_) and the standard deviation of copy number one (Std_A _and Std_B_) for each SNP.

### HMM transition distribution

The transition probability specifies the probability of changing from the MCP state at one SNP (denoted by MCP_1_) to the MCP state at the next adjacent SNP (denoted by MCP_2_). Similar to the LOH and copy number HMM [12,22], we assumed that MCP changes are caused by genetic recombination events and close SNP markers are more likely to have the same MCP, and used the Haldane's map function *θ *= (1 - e^-2D^)/2 [40] to convert the chromosomal distance D (in the unit of 100 Mb ≈ 1 Morgan) between two SNPs to the probability 2*θ *(denoted by P_0_) that MCP_2 _will return to the background MCP distribution in this sample and thus independent of MCP_1_. The background MCP probabilities (P_b_) are set non-informatively to be 0.9 for MCP of 0.5 (normal locus) and 0.1/N for the rest N MCP states. The transition probabilities are thus:

P(MCP_2_|MCP_1_) = P_0 _× P_b_(MCP_2_) if MCP_1 _≠ MCP_2_

(1-P_0_) + P_0 _× P_b_(MCP_1_) if MCP_1 _= MCP_2_.

Although Haldane's map function is traditionally used in linkage analysis to describe meiotic crossover events, the motivations of applying it here are that allelic-imbalance or copy number changes can be caused by mitotic recombination events, and mitotic recombination events may share similar initiation mechanisms and hot spots with the meiotic crossover events [41].

### HMM emission distribution

The emission distribution specifies the probability or density of the observed RAP (raw allele A proportion, defined as R_A_/(R_A _+ R_B_)) given the unobserved MCP of a SNP in a sample. If the ordered genotype alleles (G_1_, G_2_) for the minor and major parental copy in normal sample (paired or unavailable) are known, the function relating RAP to MCP and ordered genotype is:

(1) $$\text{RAP}∼\text{Normal(}\mu \text{,}\sigma^{\text{2}}\text{), and }\mu \;{\text{(G}}_{\text{1}}{\text{, G}}_{\text{2}}\text{)}=\{\begin{array}{ll} \text{1} & {\text{if (G}}_{\text{1}}{\text{, G}}_{\text{2}}\text{)}=\text{(A, A)}\\ \text{MCP} & {\text{if (G}}_{\text{1}}{\text{, G}}_{\text{2}}\text{)}=\text{(B, A)}\\ \text{1}-\text{MCP} & {\text{if (G}}_{\text{1}}{\text{, G}}_{\text{2}}\text{)}=\text{(A, B)}\\ \text{0} & {\text{if (G}}_{\text{1}}{\text{, G}}_{\text{2}}\text{)}=\text{(B, B)} \end{array}$$

For example, if the ordered genotype is (A, B) and MCP is 0.5 (a normal locus), then RAP should have a mean of 0.5; if the ordered genotype is (A, A) and MCP is 0.66 (e.g. one parental chromosome has 1 copy and another has 2 copies), then RAP has mean of 1 since both parental alleles have genotype A. The standard deviation of RAP (*σ*) has default value of 0.1, which was chosen based on empirical observation of noise level from data.

In practice, the ordered normal genotypes are only partially known in paired normal samples (as unordered genotypes) or unavailable in tumor-only samples. We averaged all the four possibilities of ordered genotype in Equation 1 to obtain the emission density function:

(2) $$\text{P (RAP}|\text{MCP)}=\sum_{{\text{(G}}_{\text{1}}{\text{,G}}_{\text{2}}\text{)}}\phi \left(\frac{\text{RAP}-\mu \;{\text{(G}}_{\text{1}}{\text{,G}}_{\text{2}}\text{)}}{\sigma }\right)\times {\text{P(G}}_{\text{1}}{\text{,G}}_{\text{2}})$$

where *ϕ *is the standard normal density function and P(G_1_, G_2_) is the probability of a ordered genotype. When the paired normal sample is available, we let P(G_1_, G_2_) be determined mainly by the observed normal genotypes and the genotyping error rate *e *(default value 0.01): P(A, A), P(A, B), P(B, A) and P(B, B) will be {1 - *e*, *e*/4, *e*/4, *e*/2} for observed genotype AA, {*e*/2, *e*/4, *e*/4, 1 - *e*} for BB, and {*e*/2, (1 - *e*)/2, (1 - *e*)/2, *e*/2} for AB. This in effect compares the RAP in a tumor sample to the genotype of the paired normal, and is similar to the LOH analysis of paired normal and tumor samples. When the paired normal sample is not available, we used the normal samples in the dataset or an independent reference set of normal samples in the same ethnic group to estimate the probability of AA, BB and AB genotypes and then convert them to P(G_1_, G_2_) similar to the above. This is effect similar to the basic HMM for tumor-only LOH inference [22]. The emission distribution through P(G_1_, G_2_) can also be flexibly extended to consider haplotype dependence of SNPs, which can cause long stretch of homozygous SNP genotypes in retention regions. Similar to LD-HMM in tumor-only LOH analysis [22], we used adjacent *N *SNPs in a tumor sample to estimate the genotype distribution of a SNP in the unavailable normal sample to improve the estimation of haplotype dependence.

### Inferring major and minor copy allele

We modified the above HMM for inferring MCP to infer the ordered genotypes (G_1_, G_2_), where G_1 _is minor copy allele and G_2 _is major copy allele. Specifically, the inferred MCP is regarded as known data, so the posterior probability (P_G_) of different (G_1_, G_2_) can be compared and the one with the largest P_G _is regarded as the inferred ordered genotype:

(3) $${\text{P}}_{\text{G}}\text{ (RAP}|{\text{(G}}_{\text{1}}{\text{,G}}_{\text{2}}\text{))}=\phi \left(\frac{\text{RAP}-\mu \;{\text{(G}}_{\text{1}}{\text{,G}}_{\text{2}}\text{)}}{\sigma }\right)\times {\text{P(G}}_{\text{1}}{\text{,G}}_{\text{2}})$$

## List of abbreviations

SNP (Single Nucleotide Polymorphism), LOH (Loss-of-Heterozygosity), CGH (Comparative Genomic Hybridization), HMM (Hidden Markov Model), TSG (Tumor suppressor gene), Q-PCR (Quantitative PCR), MCP (Major copy proportion), MCA (Major copy allele), RAP (raw allele A proportion)

## Authors' contributions

CL, WRS and MM conceived the research design. CL carried out the analysis and drafted the manuscript. RB, BAW, WW and LAG participated in the method development. All authors read and approved the final manuscript.
